# Exploring the transformative role of drone technology in advancing healthcare delivery in Africa; a perspective

**DOI:** 10.1097/MS9.0000000000001221

**Published:** 2023-09-05

**Authors:** Gbolahan Olatunji, Timilehin David Isarinade, Kokori Emmanuel, Doyin Olatunji, Nicholas Aderinto

**Affiliations:** a Department of Medicine and Surgery, University of Ilorin, Ilorin; b Department of Medicine and Surgery, Ladoke Akintola University of Technology, Ogbomoso, Nigeria; c Department of Health Sciences, Western Illinois University, Illinois, USA

**Keywords:** Africa, drone technology, healthcare delivery, innovation

## Abstract

This perspective article delves into the transformative potential of drone technology in revolutionising healthcare delivery in Africa. The continent faces numerous challenges in providing timely and efficient medical services to its vast and diverse population, compounded by geographical barriers, inadequate infrastructure and limited access to medical facilities. Amidst these challenges, the integration of drone technology emerges as a promising solution, offering unprecedented opportunities to overcome longstanding obstacles and improve healthcare accessibility across Africa. Drawing from existing drone-based healthcare initiatives in Africa, the article explores various applications of drones in healthcare delivery. These encompass but are not limited to, delivering vaccines, medications, blood samples, diagnostic tools and medical personnel to remote locations in a timely and cost-effective manner. Furthermore, the paper examines the operational challenges and regulatory considerations in deploying drone technology for healthcare and the ethical implications surrounding privacy and security.

## Introduction

Drone technology, also known as unmanned aerial vehicles (UAVs)^1^, has witnessed significant advancements in recent years and has found diverse applications across various industries. One of the most promising areas for its implementation is in healthcare, where it can bring about a revolutionary transformation in medical services worldwide, particularly in regions with limited access to healthcare infrastructure or during emergencies^2^.

Drone technology has already proven its worth in the healthcare sector through several key applications. These include the delivery of critical medical supplies like vaccines, medications, blood products, diagnostic samples and even donor organs to remote or hard-to-reach areas^3^. Furthermore, drones with medical equipment, defibrillators, or first aid kits can rapidly deliver emergency medical services^4^. Additionally, the technology facilitates telehealth services, enabling remote consultations between healthcare providers and patients in remote regions^5^. Moreover, drones with imaging technologies, such as thermal cameras or multispectral sensors, can surveillance disease during outbreaks, providing valuable data for timely medical interventions^6^.

With its vast and challenging landscapes and inadequate healthcare infrastructure in many regions, Africa presents a prime market for drone technology innovation^7^. Various initiatives have already been launched across the continent, leveraging drones to enhance healthcare services. The dense population of remote rural areas, islands and forested regions in Africa makes conventional healthcare delivery difficult, if possible. Drones offer a solution to overcome these geographical barriers by swiftly transporting medical supplies and reaching patients in need, improving access to healthcare services for all^7^.

Many African countries need help with transportation infrastructure limitations, such as poor road networks or impassable routes during certain seasons. Drones offer a more efficient and reliable medical supply delivery and emergency response mode, bypassing these challenges^2^. Research has shown the feasibility of using drones to transport blood products while maintaining appropriate storage temperatures without compromising the accuracy of routine medical analyses^8–10^. Drones have already been successfully utilised in trauma events and obstetric emergencies, such as postpartum haemorrhage, significantly impacting maternal mortality rates in Rwanda and providing blood supplies to thousands of health facilities across Ghana^11,12^.

This paper aims to shed light on the vast potential of drone technology in the healthcare sector, specifically focusing on its applications in Africa. By thoroughly examining its applications, potential and challenges in the region, the article intends to raise awareness within the healthcare industry about the benefits of embracing drones.

## Potentials of drone technology for the African healthcare system

Drone technology can potentially revolutionise the African healthcare system by addressing some of the continent’s most pressing challenges (Table 1). With their capacity to overcome geographical barriers, improve medical logistics and enhance healthcare accessibility, drones offer promising solutions to transform healthcare delivery and improve health outcomes for millions across Africa.

**Table 1:** Comparison of drone technology versus conventional transportation methods for healthcare delivery.

Parameters	Drone technology	Conventional transportation	Likely impact on healthcare delivery in Africa
Speed	Rapid delivery within minutes.	Time-consuming, depending on road	Faster delivery of medical supplies, vaccines, and medications to remote and underserved areas, leading to improved healthcare access and response during emergencies.
		conditions and distance.	
Reach	Access to remote and difficult	Limited access to remote or	Overcoming geographical barriers and reaching isolated communities, improving healthcare services in hard-to-reach regions with limited infrastructure.
	terrains.	inaccessible regions.	
Cost	Initial investment required, but	Ongoing expenses for maintenance,	Long-term cost-effectiveness, as drones can operate with reduced operational costs compared to conventional transportation methods, especially in challenging terrains.
	cost-effective in the long run.	fuel, and vehicle upkeep.	
Infrastructure requirements	Minimal infrastructure required,	Robust road and transportation	Suitable for regions with limited infrastructure, reducing the need for extensive road networks, making it ideal for remote and rural areas.
	suitable for rural areas.	networks necessary.	
Response to emergencies	Quick response for medical	Limited responsiveness in	Enhanced emergency medical services, as drones can rapidly deliver critical medical supplies, support disaster relief efforts, and improve emergency response times.
	emergencies and disaster relief.	emergency situations.	
Environmental impact	Reduced carbon footprint with	Higher emissions and pollution from	Environmentally friendly option, contributing to reduced carbon emissions and pollution, supporting sustainable healthcare practices.
	electric-powered drones.	conventional vehicles.	
Reliability	Subject to weather conditions	Subject to traffic congestion and	Weather resilience and ability to navigate challenging conditions, making drones more reliable in regions with unpredictable weather patterns.
	and technical malfunctions.	breakdowns.	

### Enhancing the medical supply chain and logistics

Delivering medical supplies to remote and underserved African areas poses significant challenges due to poor road infrastructure, long distances, difficult terrains, limited healthcare facilities, extreme weather conditions and geopolitical factors that impact healthcare access^13^. These factors are further complicated by inadequate storage facilities and unreliable transportation systems, leading to delayed access to essential medicines, vaccines, and medical aid^13^. However, drone technology presents a promising solution to overcome these challenges. Drones, also known as UAVs, have the potential to swiftly and efficiently transport medical supplies, vaccines and equipment to even the most remote and inaccessible areas^14^. By bypassing road limitations and flying over obstacles, drones can deliver essential items directly to healthcare facilities or designated drop-off points, ensuring aid reaches even the most difficult terrains.

Using drones in Africa’s medical supply chain and logistics offers several significant benefits. During emergencies and disease outbreaks, timely access to healthcare resources is crucial and drones enable rapid and reliable delivery of medical supplies, vaccines and equipment to remote and underserved areas, overcoming the limitations of traditional transportation methods^14,15^. Moreover, drones minimise the risk of stockouts and wastage of perishable medical supplies by enabling real-time inventory management and precise delivery^14^. They can transport sensitive items, such as vaccines or blood samples, under controlled conditions, ensuring their integrity and quality during transit, thus improving healthcare service delivery.

In addition to addressing logistical challenges, drones can contribute to long-term cost savings in some scenarios. Although the initial setup costs may be significant, drone delivery systems’ operational expenses can be lower than traditional transportation methods^16^. This cost-effectiveness is particularly beneficial in resource-constrained settings where healthcare budgets are limited, allowing for a more efficient allocation of resources.

### Expedited emergency response and disaster relief

Africa, alongside Asia, is one of the continent’s most vulnerable to climate-associated disasters and emergencies, including droughts, floods, wildfires, storms, heatwaves, traumatic injuries and disease outbreaks^17^. These challenges are often exacerbated by poverty, overwhelmed healthcare systems, illiteracy, poor infrastructure, difficult geographical terrain, a lack of early warning systems and governance issues^18^.

While UAVs were previously restricted to military operations, inspections, surveys and deliveries, COVID-19 opened up new possibilities in healthcare delivery^14^. Drones have been deployed to deliver medications, enforce curfews and even detect the temperatures of shoppers in major shopping centres. In crises, drones find applications in data collection, real-time information and situation monitoring, public information and advocacy, search and rescue operations, mapping, logistics and package delivery^19^. They have successfully transported microbiological samples, blood products, vaccines, medications, medical equipment and relief materials to rural and remote areas affected by crises^14,20^.

Drones also play a crucial role in assessing damage, mapping affected regions and facilitating rescue operations. They can generate geo-referenced or 3D maps that are faster and more detailed than conventional satellite imagery. This mapping helps establish early warning protocols, reduce risks, assess damage, improve logistics, conduct rescue operations and monitor situations^19^. In the aftermath of disasters such as the earthquake, cholera epidemic and Hurricane Sandy in Haiti, drones were employed to register lands, assess destroyed houses, conduct censuses of public buildings, shelters, hospitals and schools, evaluate flood and drought damage and monitor internally displaced persons and camps. This information enabled aid workers to determine when shelters were empty and camps could be closed^19^. One notable innovation, NASA’s Finding Individuals for Disaster and Emergency Response (FINDER), is equipped with ultra-sensitive sensors that can detect the heart rates and breathing patterns of individuals trapped under rubble, greatly aiding rescue operations^21^.

### Improving accessibility to remote healthcare

Various factors influence access to healthcare, broadly categorised into geographical and nongeographical factors^22^. Geographical factors consider the time and distance between health service providers and consumers, while nongeographical factors include social class, income, age, sex, health-seeking behaviour, cultural traits and knowledge of health and healthcare.

In Africa, access to healthcare services is already challenged by insufficient numbers and an imbalanced skill mix of health workers. These issues are further exacerbated by geographical barriers that hinder people from reaching the necessary care promptly^23^. The WHO recommends that everyone live within a 5 km radius or a maximum travelling time of 60 min to a health facility, using travel time as a measure of geographical accessibility^24^. However, this milestone is only achieved in some areas of Africa, and even where it is, other factors like the range of services provided at those facilities come into play. Often, primary healthcare facilities, which are more common in the region, offer only basic preventive and curative services and must be operational 24/7 or equipped with the necessary medical equipment. This leads to referrals to secondary and tertiary healthcare centres, usually farther from rural centres, creating barriers to accessing essential healthcare services^23^.

To address these challenges, drone-enabled telemedicine services have emerged as a promising solution. Drones now enable clinical monitoring of vital signs like body temperature, oxygen saturation, heart rate, virtual medical consultations and early disease detection^14,25^. They also help address challenges like insufficient beds and personal protective equipment for healthcare workers through digital communications^14,25^. By employing this technology, geographical barriers to healthcare access can be overcome, patients can be triaged efficiently, social distancing can be maintained and access to general and specialist health services can be improved. Additionally, drones can play a crucial role in tackling the many challenges posed by inadequate physical healthcare infrastructure in the region^24,26^.

### Efficient disease surveillance

Early detection of disease outbreaks is crucial for timely intervention and mitigating the impact on public health. Effective disease surveillance and notification systems play a vital role in detecting and monitoring diseases and other events that pose potential threats to public health regarding their source, timing, affected individuals, populations and locations, forming the basis for public health actions^27^.

Drones with high-altitude life video streams, imaging technologies and sensors offer rapid data collection and monitoring capabilities, providing a quick overview of conditions. They prove helpful in assessing the damage caused by disasters, such as collapsed buildings, bridges, or blocked roads and tracking the distribution of relief materials like tents, medical equipment and vaccines. Furthermore, drones can monitor the movement of people to and from temporary settlements, aiding in humanitarian crisis management^14,19^. Medical screenings, including body temperature, oxygen saturation and breathing rate assessments from a distance, are also facilitated by sensors attached to drones. Algorithms designed for understanding human movements can help identify potential symptoms like coughing or sneezing, which are relevant in epidemiological nursing^14,25^.

Data collected through drones’ use in epidemiological surveillance are important for real-time analysis and targeted interventions, enabling proactive disease management and crisis response. The fusion of sophisticated technological innovation with immediate response capabilities allows drones to intervene with high precision during natural disasters and emergencies^20^. For instance, drones played a crucial role during Super Typhoon Haiyan’s devastation of Tacloban, Philippines, in November 2013. They were deployed to identify suitable locations for operation bases and assess the passability of roads, tasks that could have taken days if done on foot or by helicopter. Additionally, drones were flown along the coast to assess damage from storm surges and flooding, providing crucial information for response efforts. Their aerial assessments accelerated response times, reduced unnecessary work and improved the accuracy and precision of assistance rendered. In the event of arriving within 72 h, drones might have also located survivors in the rubble using infrared cameras^19^. This experience highlights the immense potential of efficient surveillance using drone technology in healthcare delivery during disasters and emergencies.

### Overcoming infrastructural challenges

Despite its wealth of resources, Africa faces significant challenges due to limited infrastructure, including poor roads, inadequate transportation systems and remote regions with difficult access^3^. These limitations have considerably strained healthcare accessibility, especially in rural and impoverished areas, resulting in obstacles to equitable healthcare access. Infrastructural barriers make it difficult for patients to reach hospitals for medical help or essential procedures, leading them to either neglect their illnesses or resort to traditional and unconventional remedies, often with negative consequences for their health. Implementing drones in healthcare can effectively address these challenges by providing a reliable and efficient mode of transportation, bypassing infrastructural limitations and ensuring timely access to medical services^7^.

Drones offer cost-effective solutions for transporting medical supplies and samples in Africa. Unlike traditional transportation methods, which are often expensive and slow, drones can navigate difficult terrains, deliver supplies directly to remote locations and operate at a fraction of the cost. Additionally, drones can transport small, lightweight payloads, reducing the need for large and costly transportation infrastructure while contributing to environmental preservation by lowering vehicle carbon emissions. By optimising logistics and reducing costs, drones can enhance the efficiency and affordability of healthcare delivery in resource-constrained settings^28^.

Drone technology presents a unique opportunity to achieve equitable access to healthcare in Africa without the need for extensive infrastructure development. Drones can access remote and inaccessible areas, overcoming geographical barriers often hindering traditional healthcare delivery^3^. By establishing drone networks, medical supplies, vaccines and diagnostic samples can be swiftly and efficiently transported, ensuring that even the most isolated communities can access essential healthcare services. This approach eliminates the need for extensive road networks or costly healthcare infrastructure investments, leading to budget reductions. Instead, drones offer a scalable and adaptable solution that can be rapidly deployed, benefiting underserved populations and promoting equitable access to healthcare throughout Africa.

## Challenges and considerations for implementation

**Figure 1:**
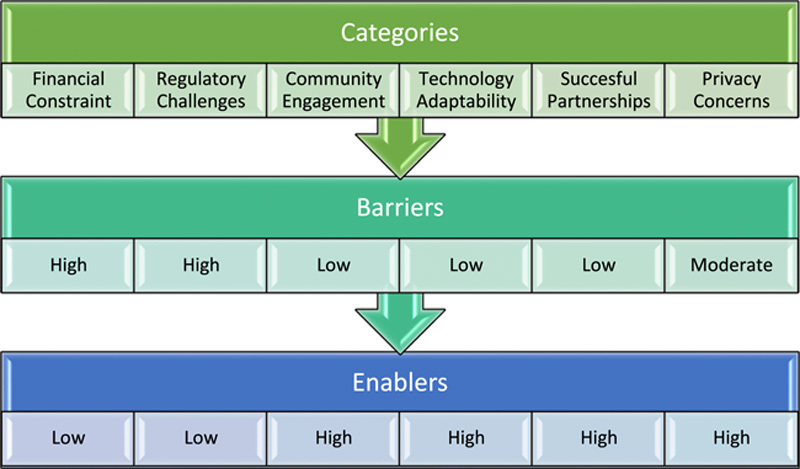
Barriers and enablers for drone-based healthcare delivery in Africa.

Drones indeed hold immense potential in transforming healthcare delivery, offering innovative solutions to longstanding challenges (Fig. 1). However, their seamless integration into healthcare systems across Africa faces several hurdles, with the regulatory framework being a significant obstacle. Aviation authorities, such as the Federal Aviation Administration (FAA)^29^, have implemented regulations to ensure the safe operation of drones. These primarily focus on avoiding collisions with manned aircraft, maintaining visual line-of-sight during flights and restricting flights over populated areas. While these regulations are essential for overall safety, they may only partially be conducive to the unique requirements of African medical drone operations.

To unlock the full potential of medical drones in the African healthcare landscape, it is imperative to adapt existing regulations to suit the specific needs and realities of the region. This involves fostering collaboration between aviation authorities, healthcare stakeholders, technology developers and local communities to formulate guidelines that balance safety and flexibility. Emphasising operational protocols that cater to Africa’s diverse and remote geographical settings will be crucial to ensuring the successful implementation of medical drone services.

Additionally, addressing the concerns of privacy, confidentiality and data protection is paramount when employing medical drones in healthcare delivery. As drones capture and transmit sensitive medical information, compliance with relevant patient confidentiality laws, such as the Protection of Personal Information Act (POPIA) in South Africa^30^, becomes vital. Nigeria’s Patients’ Bill of Rights also underscores the need for safeguarding patient data^31^. Healthcare institutions and drone operators must prioritise robust data encryption, secure communication channels and clear guidelines for handling and storing protected health information. Moreover, transparent communication with patients and communities about data usage and consent procedures will foster trust and confidence in medical drone services.

Safety remains a top priority in successfully integrating medical drones into healthcare systems. Technical challenges, such as malfunctions, cyber threats and reliance on GPS technology, must be diligently addressed to mitigate potential risks^32^. Collaborative efforts among relevant stakeholders are needed to establish comprehensive air-traffic management systems, implement real-time monitoring of drone operations and develop standardised safety procedures for medical drone flights. Furthermore, continuous training and certification programs for drone operators will enhance their expertise and confidence in handling diverse situations, ensuring a safe and reliable drone healthcare network.

Moreover, African countries’ climatic and geographical diversity poses further challenges to drone operations^33^. Adverse weather conditions and limited visibility can significantly impact drone performance and reliability. To mitigate these challenges, extensive research must understand how drones function under varying weather and daylight situations. Investing in communication networks and infrastructure will also be crucial to ensure seamless telecommunication capabilities, even in remote areas and ensure continuous operational availability of medical drones.

The successful adoption of medical drones depends on overcoming technical and regulatory barriers and addressing community acceptance and perception. Public awareness and education about the benefits of medical drones and their potential to improve healthcare access and outcomes will be essential^34^. However, it is equally crucial to acknowledge and address concerns related to privacy, noise pollution and the potential misuse of drone technology. Engaging with local communities, healthcare providers and regulatory authorities to gather feedback and incorporate their perspectives in the planning and implementation will foster a sense of ownership and acceptance.

Despite the potential benefits, drone transportation costs can sometimes outweigh conventional means. Limited financial resources and sustainable financing models pose challenges in many African countries^35,36^. International partnerships, as exemplified by the collaboration between the Rwandan government and Zipline, a U.S. firm, have successfully addressed infrastructure challenges and enabled the implementation of drone-based healthcare systems. Such partnerships are instrumental in expanding drone-based healthcare to other nations in sub-Saharan Africa.

## Conclusion

Drone technology presents a transformative opportunity for revolutionising healthcare in Africa. By addressing challenges related to accessibility, medical supply chains, emergency response and disease surveillance, drones can bridge the gap between remote areas and healthcare services, ensuring equitable access to medical care. The collaboration between governments, healthcare providers and technology companies will be pivotal in harnessing the full potential of drones to improve healthcare delivery and create a healthier future for the continent. By integrating drones into healthcare systems, Africa can overcome infrastructural limitations, reduce delivery times and enhance disaster relief efforts, improving health outcomes and overall healthcare accessibility for its population.

## Ethical approval

Ethical approval is not applicable for this perspective.

## Consent

Informed consent is not applicable for this perspective.

## Sources of funding

No funding was received for this perspective.

## Author contribution

N.A.: conceptualization. All authors contributed in writing of first and final draft.

## Conflicts of interest disclosure

All authors declare no conflicts of interest.

## Research registration unique identifying number (UIN)

1. Name of the registry: not applicable.
2. Unique identifying number or registration ID: not applicable.
3. Hyperlink to your specific registration (must be publicly accessible and will be checked): not applicable.

## Guarantor

Nicholas Aderinto.

## Data availability statement

No new dataset were generated for this perspective.

## Provenance and Peer Review

Not commissioned, externally peer-reviewed.
